# Adamantinoma of the distal femur diagnosed 5 years after initial surgery: a case report

**DOI:** 10.1186/s13256-016-0974-8

**Published:** 2016-06-23

**Authors:** Kai Cao, Michiro Susa, Itsuo Watanabe, Kazumasa Nishimoto, Keisuke Horiuchi, Aya Sasaki, Yuichiro Hayashi, Katsura Emoto, Kaori Kameyama, Masaya Nakamura, Morio Matsumoto, Hideo Morioka

**Affiliations:** 1 Department of Orthopedic Surgery, Keio University School of Medicine, 35 Shinanomachi, Shinjuku-ku Tokyo, 160-8582 Japan; 2 Department of Orthopedic Surgery, the First Affiliated Hospital of Nanchang University, Nanchang, 330006 China; 3 Division of Surgical Pathology, Keio University School of Medicine, 35 Shinanomachi, Shinjuku-ku Tokyo, 160-8582 Japan

**Keywords:** Adamantinoma, Metastatic adamantinoma, Medial femoral condyle, Distal femur

## Abstract

**Background:**

Adamantinoma arising in the femur is extremely rare. We report a case of an adamantinoma occurring in the right medial femoral condyle that was diagnosed 5 years after the primary surgery.

**Case presentation:**

A 74-year-old Asian woman first complained of right knee pain without any cause. Radiographs demonstrated a 4×4.5 cm osteolytic lesion in her medial femoral condyle. Magnetic resonance imaging revealed a lesion which showed low signal on both T1 and T2-weighted image, and enhanced signal with gadolinium contrast administration. She underwent a wide resection of the lesion and was reconstructed with a tumor endoprosthesis. On histological examination, the tumor showed clusters of spindle-shaped and squamoid epithelial cells among the fibrous stroma. Adamantinoma was considered, however, the diagnosis was inconclusive due to the unusual localization and her age. Moreover, it was difficult to exclude metastatic carcinoma. Five years later, she was diagnosed with an abnormal shadow occupying the upper lobe of her right lung in a routine physical examination. She subsequently underwent a resection of the lung mass which histologically showed proliferation of spindle-shaped and squamoid epithelial cells. The histological similarity of the lung tumor and the femoral tumor led to the diagnosis of adamantinoma arising in her right medial femoral condyle with metastasis to the upper lobe of her right lung.

**Conclusion:**

In this case report, we report the clinical, radiographic, and histological features of an adamantinoma arising in the distal femur with a review of the literature.

## Background

Adamantinoma is a malignant biphasic tumor characterized by a variety of morphological patterns, most commonly clusters of epithelial cells, surrounded by a relatively bland spindle osteofibrous component. Adamantinoma comprises approximately 0.4 % of all primary bone tumors. It usually arises in the center of long bones, and 97 % of all reported cases occur in long tubular bones [1, 2]. The tibia, in particular the anterior metaphysis or diaphysis, is involved in 85 to 90 % of cases. Among other long bones, fibula and ulna are rarely affected [1, 2]. Clinical symptoms such as swelling and radiographic abnormality may last for many years before definitive diagnosis is made because of the difficulty of diagnosis at the referral time [2–4]. Although classic adamantinomas are easily recognizable with characteristic epithelial and osteofibrous components, in some circumstances, small clusters of epithelial cells are the only clue for a definitive diagnosis.

We report an unusual case of adamantinoma of the medial femoral condyle finally diagnosed 5 years after initial surgery. To the best of our knowledge, this is the first reported case of an adamantinoma arising in the distal femoral condyle.

## Case presentation

**Fig. 1:**
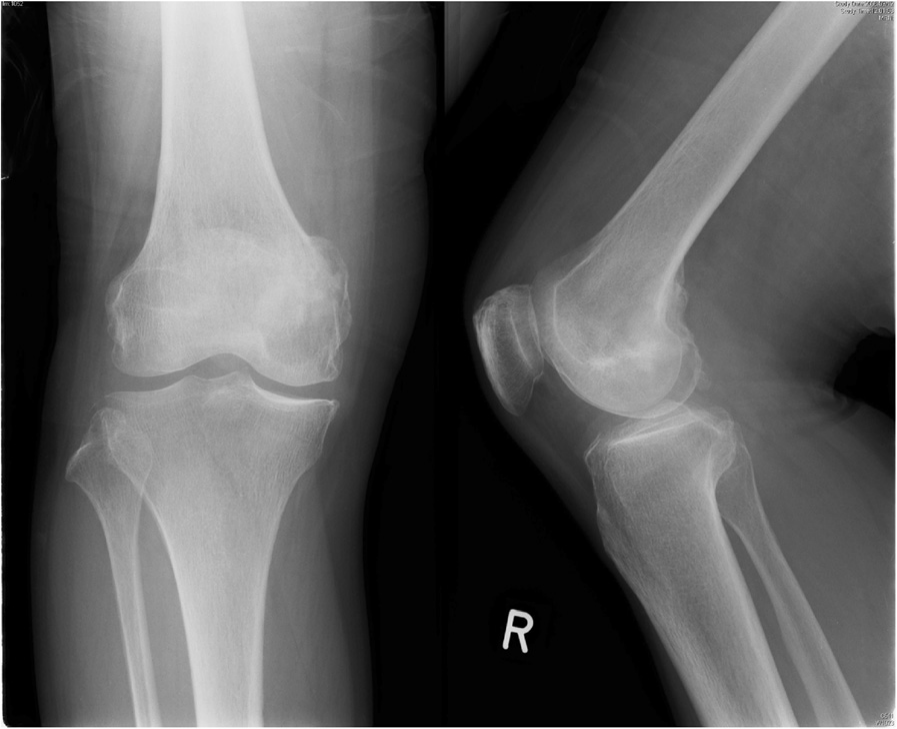
Plain radiographs of the tumor in the distal femur. Anteroposterior and lateral radiographs show a 4×4.5 cm osteolytic lesion in the right medial femoral condyle

**Fig. 2:**
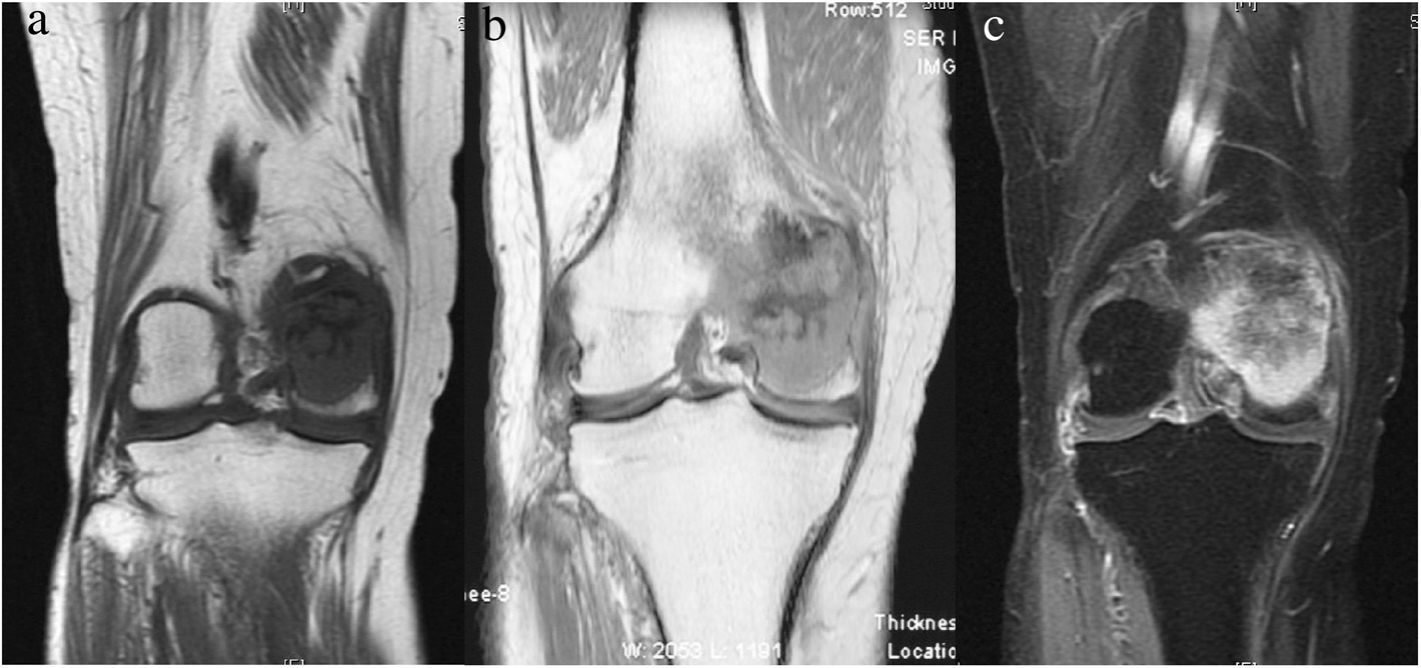
Magnetic resonance imaging of the tumor in the distal femur. On magnetic resonance imaging, the tumor was depicted as a low signal lesion on T1-weighted image (**a**), low to intermediate intensity on T2-weighted image (**b**), and highly enhanced with gadolinium contrast (**c**). There was no apparent destruction of the cortex and no soft tissue mass was present

**Fig. 3:**
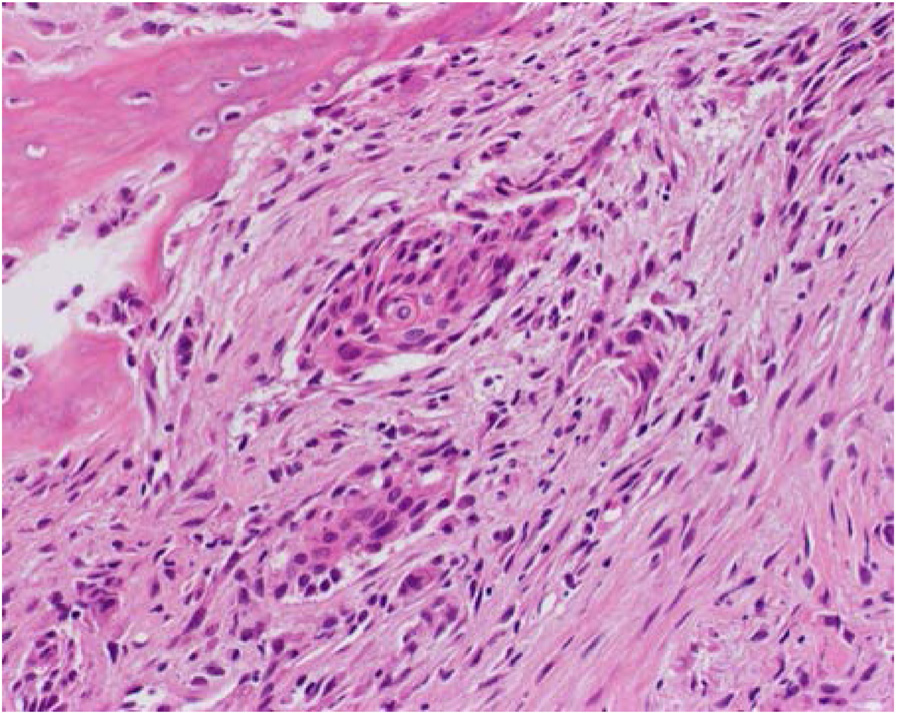
Histopathology of the femoral lesion. The tumor shows clusters of epithelial cells that were oval or spindle-shaped with squamous differentiation, and surrounded with fibrous stroma. Tumor cell nuclei are relatively uniform, did not show pronounced atypia, and have a low rate of mitosis (hematoxylin and eosin)

**Fig. 4:**
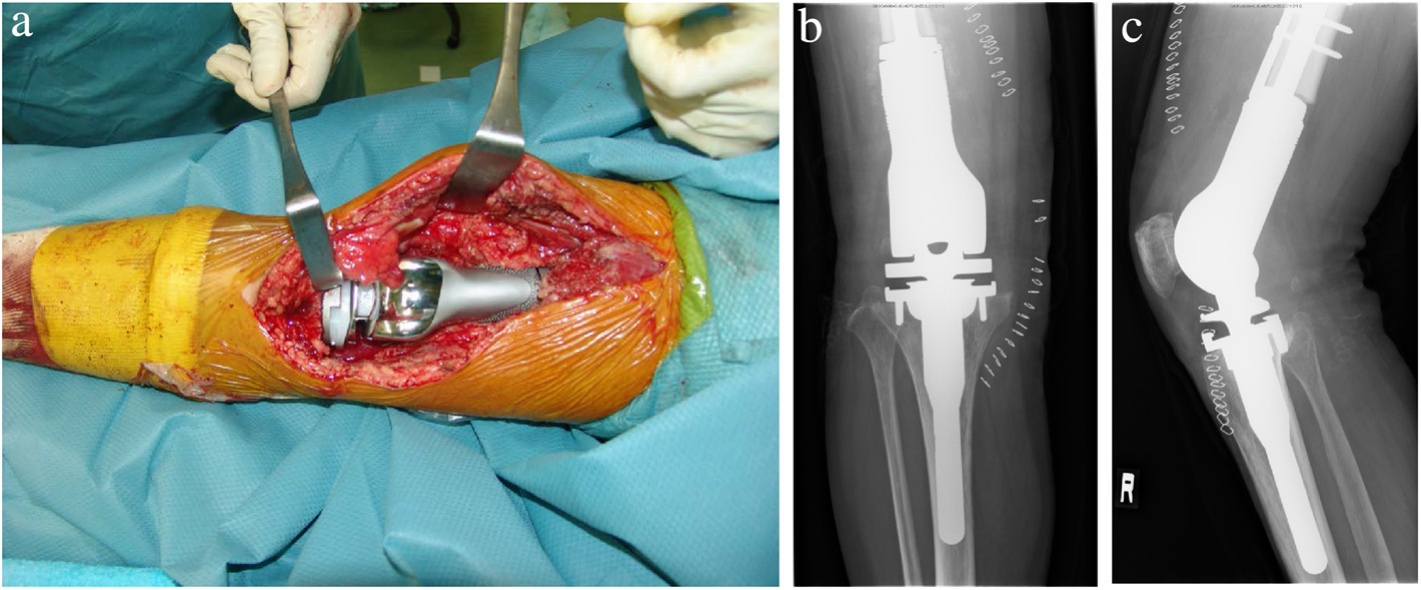
Intraoperative finding. Wide resection of the tumor was performed and was reconstructed with a tumor endoprosthesis (**a**). Anteroposterior (**b**) and lateral (**c**) radiograph after operation

**Fig. 5:**
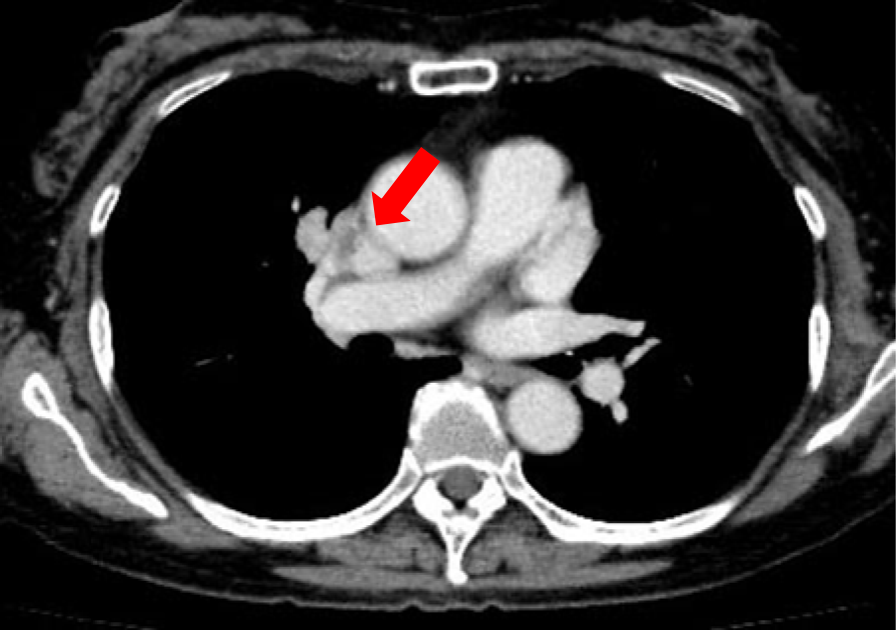
Computed tomography imaging of the lung. Five years after initial surgery, the patient took a routine physical examination including chest computed tomography which revealed an abnormal shadow occupying her right upper lung (*arrow*)

**Fig. 6:**
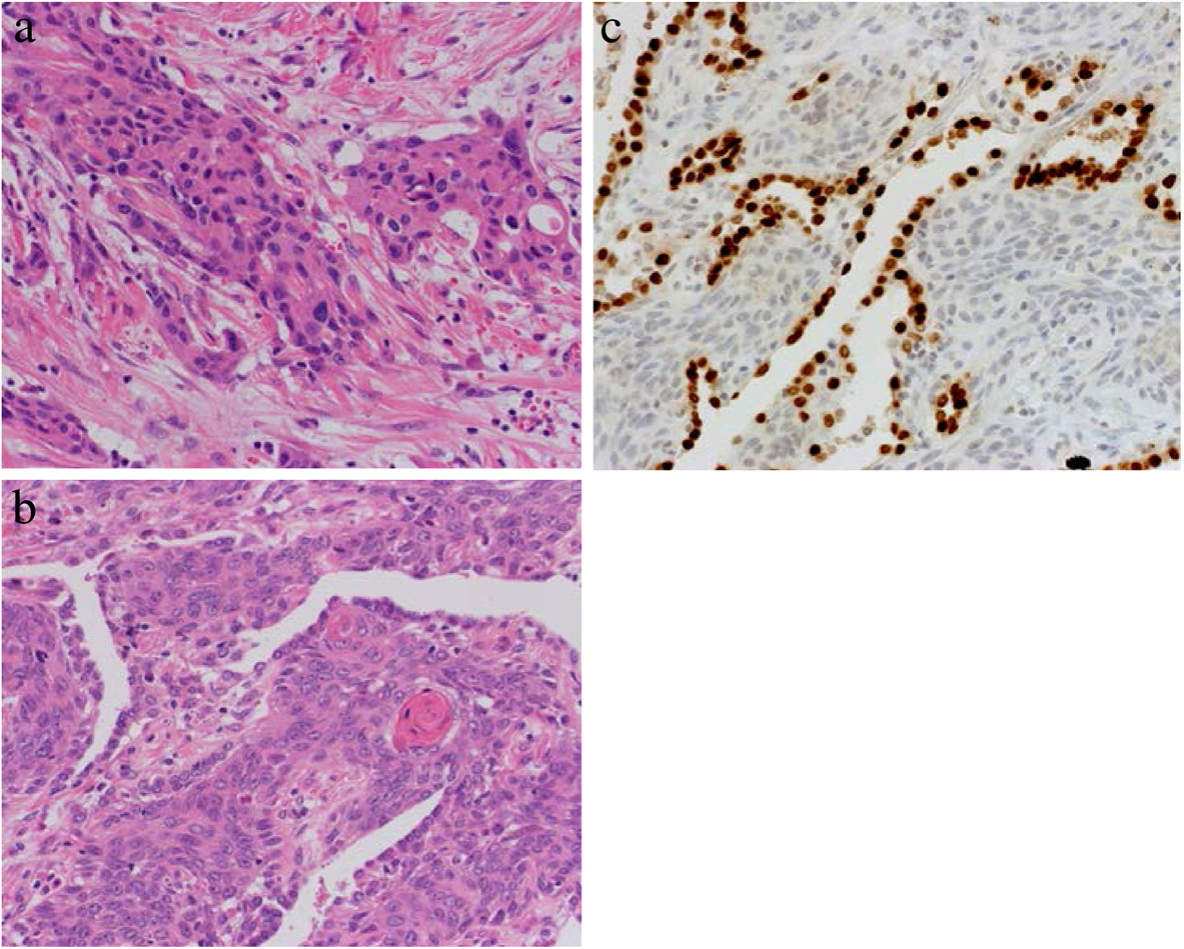
Histopathology of the upper lung lesion. The tumor shows proliferation of spindle-shaped and squamoid epithelial cells with mild nuclear atypia, surrounded with fibrous stroma, which is strikingly similar to the histopathological findings of the femoral lesion (**a**). The epithelial cells spread around the alveolar wall without destruction of the alveolar structure (**b**). Immunostaining of thyroid transcription factor-1 demonstrates preserved epithelial cells of alveolar wall. The tumor cells are negative for thyroid transcription factor-1 (**c**)

A 74-year-old Asian woman complained of right knee pain without any cause. Because the pain persisted for several months, she went to a nearby hospital where she was referred to our institute for a second opinion and treatment. She had thyroid cancer and received tumor resection 5 years prior to the knee symptom without local recurrence. Radiographs demonstrated a 4×4.5 cm osteolytic lesion in her medial femoral condyle (Fig. 1). On magnetic resonance imaging (MRI), the lesion was depicted as low intensity on T1-weighted image (T1WI), low-intermediate intensity on T2-weighted image (T2WI), and highly enhanced after gadolinium contrast administration. There was no penetration of the tumor through the cortex and no soft tissue mass was present (Fig. 2a–c). An open biopsy was performed and, histologically, the tumor showed clusters of epithelial cells. These epithelial cells were oval or spindle-shaped with squamous differentiation, and surrounded with fibrous stroma. Tumor cell nuclei were relatively uniform, did not show pronounced atypia, and had a low rate of mitosis. Reactive bone formation, similar to fibro-osseous lesion, was seen around the epithelial cells (Fig. 3). On histological examination, adamantinoma was initially considered for the diagnosis. However, it was inconclusive due to its unusual localization. Considering the patient’s age, metastatic squamous cell carcinoma was possible although the primary lesion was not evident. Moreover, it was difficult to exclude metastasis of the thyroid cancer she had 5 years ago because papillary carcinoma of the thyroid gland sometimes undergoes squamous metaplasia thereby resembling squamous cell carcinoma. Because there was no other known lesion after further screening, we performed a wide resection of the tumor and implanted a tumor endoprosthesis to reconstruct the defect (Fig. 4a–c). The resected specimen showed the same histological feature as the biopsy sample; therefore, the final diagnosis remained inconclusive. She was discharged without any complication. Five years later during a routine follow-up examination, an abnormal shadow occupying the upper lobe of her right lung was detected (Fig. 5). She subsequently underwent lobectomy for the lesion. A histopathological section showed proliferation of spindle-shaped and squamoid epithelial cells with mild nuclear atypia, surrounded by fibrous stroma, which was strikingly similar to the findings of the femoral lesion resected 5 years ago (Fig. 6a). Of interest, the epithelial cells spread through the alveolar wall without destruction of the alveolar structure. This feature is quite different from that of squamous cell carcinoma, either primary or metastatic, which usually shows destructive growth (Fig. 6b, c). The bone and lung specimen were both negative for thyroid transcription factor-1 (TTF-1) and thyroglobulin immunostain, which precluded the diagnosis of thyroid papillary carcinoma. These findings led to a conclusion that the lung mass is a metastasis from the distal femoral lesion, and the tumor which occurred in the medial femoral condyle was finally diagnosed as adamantinoma.

**Fig. 7:**
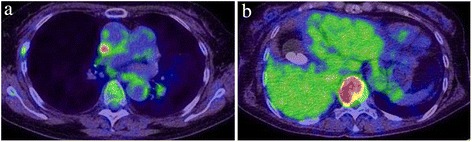
Positron emission tomography-computed tomography findings at the latest follow-up. Fluorodeoxyglucose uptake suggested a recurrent tumor in the right upper lobe of the lung (**a**) and a metastasis to the spinal vertebrae (**b**)

At the latest follow-up, positron emission tomography-computed tomography (PET-CT) showed a recurrent tumor in the upper lobe of her right lung and further metastasis in her lumbar spine (Fig. 7).

## Discussion

Adamantinomas commonly involve the tibia, fibula, and ulna. Adamantinomas can also be in other long bones including femur, humerus, and radius, but they are rarely affected [1–3]. There are sporadic case reports of adamantinoma of the rib, spine, calcaneum, metatarsal, and carpal bones [1, 5]. To the best of our knowledge, there have been 14 case reports of adamantinomas arising in the femur [2–4, 6, 7].

Keeney *et al*. reported a series of adamantinomas of long bones and six cases (7.1 %) were in the femur [2]. Five cases arose in the diaphysis of the femur and one distribution was unknown. Ii *et al*. described one lesion located in the lesser trochanter of the proximal femur which developed a pathological fracture [3]. Of the 32 adamantinomas reported by Hazelbag *et al*., only one case arose in the diaphysis of the femur [4]. Ramaswamy *et al*. reported on a recurrent adamantinoma at the right femoral diaphysis after an above knee amputation of left leg for adamantinoma of tibia [6]. Another five cases of femoral adamantinomas were reported before 1976 and the precise locations were not available [7]. To date, all the reported lesions in the femur are located either in the proximal femur or in the diaphysis of the femur. To the best of our knowledge, this is the first reported case of an adamantinoma arising in the femoral condyle.

On radiological examination, the tumor is typically well surrounded, cortical, lobulated, and osteolytic. Lucency, septation, and peripheral sclerosis may also be seen intralesionally [8]. The lesion commonly remains intracortical and extends longitudinally, but may also breach the cortex and invade the medullary cavity or soft tissue, which is usually accompanied by lamellar or solid periosteal reaction [9]. MRI is useful to detect multicentricity, extension of the lesion, and possible soft tissue involvement [10]. When tumors occur in the femur, all reported cases that had the necessary information were predominantly situated in the medullary cavity and not in the cortex with an osteolytic feature leading to a misdiagnosis. Radiographs of our case also revealed an osteolytic lesion within the medullary cavity of the femoral condyle with cortical thinning, but, no evidence of a pathological fracture.

On histological examination, classic adamantinoma is composed of an epithelial and osteofibrous component mixed in various proportions and different patterns. The four main patterns are basaloid, tubular, spindle, and squamous. Although the first two patterns are not uncommon, an admixture of all these patterns may also be seen. Basaloid and spindled variants may have a more aggressive behavior. The spindle cell component is more often observed in recurrences and in metastases [9, 11, 12]. A fifth histological pattern, the so-called osteofibrous dysplasia-like variant, in which the osteofibrous tissues are intermingled with small groups of epithelial cells, has been also described [9, 13]. In some cases, it is difficult to differentiate adamantinomas from other benign lesions, such as fibrous dysplasia or osteofibrous dysplasia. In these circumstances, a small cluster of epithelial cells may be the only feature to differentiate between benign and malignant lesions. Because of the similarity in anatomic site, radiographic features, and histologic features, some believe that adamantinoma is a malignant variant of osteofibrous dysplasia [4]. Trisomies 7, 8, 12, and/or 21 have been reported in both osteofibrous dysplasia and adamantinoma [14]. Maki and Athanasou recently investigated the relationship between adamantinoma and osteofibrous dysplasia by using immunohistochemistry to analyze the expression of several proto-oncogene products and extracellular matrix proteins [15]. A number of oncoproteins such as c-fos and c-jun were found to be commonly expressed, but differential expression of osteonectin, osteopontin, and osteocalcin between adamantinoma and osteofibrous dysplasia suggested its usefulness in distinguishing the two lesions.

The reasons that led to a late diagnosis of adamantinoma in our case may be attributed to two aspects. First, 85 to 90 % of adamantinoma cases arise in the tibia, and femur is an uncommon location for this rare tumor. Second, our patient’s past history of thyroid carcinoma provoked the possibility that the epithelial cells may be metastasis with squamous metaplasia. Considering her age, the diagnosis of metastatic carcinoma seemed to be more reasonable than adamantinoma arising in the femur, although a thorough examination revealed no possible lesion. Because of these difficulties, a definitive diagnosis was not possible until comparison with the lung lesion 5 years later. The resected lung tumor exhibited a strikingly similar histology to the initial tumor that arose in her femur and, most importantly, the tumor cells spread without the significant destructive growth pattern that is usually seen in squamous cell carcinoma. Based on these observations, the initial tumor which occurred in her medial femoral condyle was finally diagnosed as an adamantinoma.

Wide resection is recommended for the treatment of classic adamantinoma, which was associated with lower risk of local recurrence [16]. Radiotherapy and chemotherapy have not shown any encouraging results [1]. Marginal resection and histological subtype (osteofibrous dysplasia-like) were associated with a high risk of metastasis. When inadequate resection is performed, the incidence of local recurrence has been reported to be as high as 90 % [4]. In the present case, our patient underwent an initial wide resection of the distal femur without local recurrence or metastases for 5 years. However, the biological behavior of an adamantinoma is highly unpredictable as presented in our case. Lung and spinal metastasis occurred after latency, and she is currently undergoing a palliative treatment with radiation and bisphosphonate.

## Conclusions

We described a rare case of an adamantinoma of the medial femoral condyle. Adamantinoma is sometimes mistaken for a benign lesion and it is imperative to undertake a meticulous histological examination to detect any epithelial cell components especially when the lesion is located in an unusual place like the femur. Because of the unpredictable biological behavior of an adamantinoma, long-term follow-up is warranted.

## Abbreviations

MRI, magnetic resonance imaging; PET-CT, positron emission tomography-computed tomography; T1WI, T1-weighted image; T2WI, T2-weighted image; TTF-1, thyroid transcription factor-1
